# Obesity: a systematic review on parental involvement in long-term European childhood weight control interventions with a nutritional focus

**DOI:** 10.1111/obr.12046

**Published:** 2013-06-04

**Authors:** J J Kruk, F Kortekaas, C Lucas, H Jager-Wittenaar

**Affiliations:** 1 Hanze University of Applied Sciences, Professorship in Health Care and NursingGroningen, The Netherlands; 2 Fleur Kortekaas HealthAlmere, The Netherlands; 3 Department of Clinical Epidemiology, Biostatistics and Bioinformatics, University of Amsterdam, Academic Medical CentreAmsterdam, The Netherlands

**Keywords:** Childhood, nutrition, obesity, parental involvement

## Abstract

In Europe, about 20% of children are overweight. Focus on parental responsibility is an effective method in weight control interventions in children. In this systematic review we describe the intensity of parental involvement and behaviour change aimed at parents in long-term European childhood weight control interventions. We include European Union studies targeting parents in order to improve children's weight status in multi-component (parental, behaviour change and nutrition) health promotion or lifestyle interventions. The included studies have at least one objectively measured anthropometric outcome in the weight status of the child. Parental involvement was described and categorized based on the intensity of parental involvement and coded using a validated behaviour change taxonomy specific to childhood obesity. Twenty-four studies were analysed. In effective long-term treatment studies, medium and high intensity parental involvement were identified most frequently; whereas in prevention studies low intensity parental involvement was identified most frequently. Parenting skills, generic and specific to lifestyle behaviour, scored frequently in effective weight control interventions. To list parental skills in generic and specific to lifestyle, descriptions of the included studies were summarized. We conclude that intensity of parental involvement and behaviour change techniques are important issues in the effectiveness of long-term childhood weight control interventions.

## Introduction

Childhood overweight and obesity are a serious public health problem in Europe. In Europe, about 20% of children (aged 0–16) is currently overweight, of which a third is obese. This percentage represents 15 million school children. Prevalence among infants and preschoolers (aged 0–5) is high and of epidemic proportion, ranging from 12% in eastern European countries to 33% in Mediterranean countries. Over 60% of children who are overweight before puberty will be overweight in early adulthood 1–2. Childhood obesity has adverse psychological, social and health consequences in childhood and later in life 3. Children have 10 times higher risk for obesity when both their parents are obese 4. Additionally, it is known that energy balance related behaviour such as dietary behaviour is established and set before the age of 5 5–6. Experts in the area of childhood obesity recommend that prevention and treatment of obesity in the formative pre- and primary school years should focus on parents 7.

Parental beliefs, attitudes, perceptions and behaviour appear to have significant impact on the development of early overweight 4–11. Greater parental involvement and making parents responsible for participation in and implementation of lifestyle changes are identified as effective techniques in the prevention and treatment of obesity and being overweight in children 9,12. To study the effectiveness of interventions based on behaviour change processes and techniques in the programme contents, a behaviour change taxonomy was developed and validated 14. Golley *et al*. specified this taxonomy further for childhood obesity and studied short-term effectiveness of parental involvement in lifestyle interventions including a nutrition or activity component and a behavioural change component in worldwide studies that included children aged 1–18 years. Intervention effectiveness was favoured when behaviour change techniques (BCTs) spanned the spectrum of behaviour change process 9. However, the long-term effectiveness of parental involvement in these interventions remains unclear. Therefore, in this review, we aimed to describe the intensity of parental involvement and behaviour change processes and techniques aimed at parents in long-term childhood weight control lifestyle interventions, with a focus on nutritional components, in children in the age of 0–12 years in the European Union.

## Methods

### Criteria for considering studies for review

#### Inclusion criteria

Published and unpublished (non-) randomized controlled trials (RCTs), clinical controlled trials, pilot studies and observational trials and reports were eligible for inclusion in this systematic review. Restriction to RCT designs might compromise the types of parental involvement implemented in present interventions.

Intervention participation involved at least one parent or caregiver, with their child(ren). Interventions were included if they had a parental component, a behavioural change component and a nutritional component. For the multiple components, we formulated the following definitions. A parental component is an intervention with the parents or caregivers as key participants. Parents were considered key participants if the reviewers were able to identify direct parental exposure to intervention; identify active parental participation and identify active use of parenting skills in lifestyle behaviours including dietary and physical behaviour. We defined a behavioural change component as a theory-based behavioural change or well-reported method of behaviour change. A nutritional component was defined as targeting a single or multiple dietary change through education in a group or by individual counselling. The programme settings were (pre)school-based, community-based, day-care based or clinic outpatient setting.

Only trials performed in the European Union were considered for review. Trials with interventions aimed at the primary, secondary, tertiary prevention and treatment of weight control (stabilization of weight and weight loss) were included. Since we were specifically interested in parental involvement in intensive lifestyle interventions, trials were included if the duration of the interventions was 10 weeks minimum. Trials with a primary prevention focus needed to have a follow-up of at least 1 year from the start of the trial. Trials of weight control interventions needed to have a follow-up of at least 6 months after the end of the intervention was performed. Studies with at least one objectively measured (not self reported) anthropometric outcome in the weight status of the child – body mass index (BMI), BMI-standard deviation scores (BMI *z*-scores) or percentage overweight of the child – at end of intervention or interim, and/or ≤1 year post-intervention and/or ≥2 years post-intervention were included.

#### Exclusion criteria

Studies focusing on a single intervention component and studies in which weight status was not reported as an outcome variable were excluded. Furthermore, studies on obesity resulting from eating disorders, and studies in which pharmaceuticals were used as an intervention for the treatment of obesity were excluded. Trials on obesity in a specific subgroup with non-obesity related comorbidity were excluded as well.

### Search methods for identification of studies

The search was carried out in April 2011. Interventions published between January 1996 and April 2011 were searched. Search strategies and keywords for the different electronic databases were developed and assessed by both researchers and specified with the help of an information specialist. The search was performed using the following electronic databases in a systematic, structured and reproducible way: Medline (via Ovid); Embase; Psych INFO; CINAHL; the Cochrane Library: Central; Database of Abstracts of Reviews of Effects; Database of Promoting Health Effectiveness Reviews; and Health Technology Assessment. The Science Citation Index expanded and Social Sciences Citation Index Web of Science (Conference Proceedings Citation Index) were used as well. In addition, a search in the OpenSIGLE (System for Information on Grey Literature in Europe) was performed. To search for specific Dutch interventions, all lifestyle interventions of the database ‘Gezond leven loket’ of the National Institute for Public Health and the Environment (RIVM) were electronically assessed and a search in the electronic database of the Dutch Journal of Medicine (NTvG) was performed. Language restrictions were applied to English, German and Dutch.

For included studies, if parental involvement was reported only briefly, we searched for publications of the same study that provided more information on parental involvement or requested more information from the authors. In addition, reference lists of all retrieved articles and review articles were screened for potentially eligible articles. Furthermore, a number of websites of research groups that conduct and publish systematic reviews, websites and contents of programme details were systematically searched, with a key focus on Dutch websites and programmes. The International Standard Randomised Controlled Trial Number register, the register of the National Institute of Health (http://www.controlled-trials.com) and the Dutch Trial register (http://www.trialregister.nl) were searched for ongoing trials. All authors of ongoing trials were contacted for details on unpublished results. The 2010 report of the European network for public health, health promotion and disease prevention EuroHealthNet was screened for obesity prevention and treatment programmes 15.

#### Selection of studies

**Figure 1:**
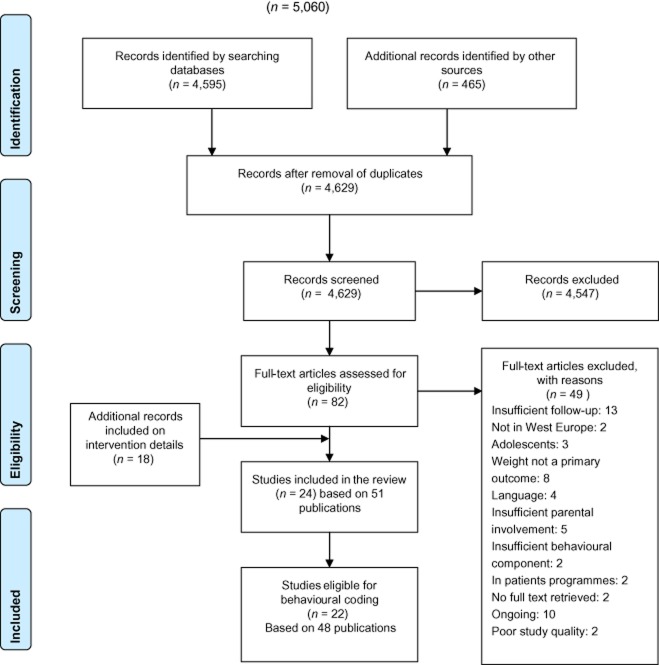
Search results: 2009 PRISMA statement flow diagram: interventions involving parents in child weight control studies 16.

After performing the search strategy described previously, the second author (FK) assessed the records retrieved by the database search and selected full texts for eligibility, based on title and abstract. Review criteria were further specified based on the records retrieved. Two authors (JvdK, FK) independently assessed these full texts to identify studies meeting the inclusion criteria, using a self-developed form. Discrepancies were resolved between reviewers by reaching consensus. In case of discrepancies between the authors, final resolution by a third party arbitrator (CL) was made. In Fig. 1 the search results are visualized.

### Data extraction and synthesis

Two reviewers (JvdK, FK) performed data extraction independently, using piloted and standardized coding forms. The data collection included a description of the parents' involvement, a categorizing of the intensity of parental involvement, a list of reported BCTs and a summary of results of primary outcomes. Moreover, only those aspects of the interventions which were aimed at the parent(s) and child were coded. Only the intervention under study was coded. Studies categorized with unclear parental involvement were not coded. The methodological quality of included studies was assessed by two reviewers independently (JvdK, FK) using the CONSORT statement with the extension for non-pharmacologic treatment (NPT) interventions 17. The risk of bias was assessed as instructed in the Handbook of the Cochrane review group 18. Discrepancies were resolved between the reviewers by consensus. Studies with a low quality rating or high risk of bias were excluded from the review.

Methods of parental involvement were described and categorized based on the intensity of parental involvement: high involvement, medium involvement, low involvement or unclear involvement 19. The intensity of parental involvement was measured scoring the time the healthcare provider spent in direct contact with the parents and the frequency of contact moments. High involvement was defined as parents are directly involved in multiple activities or in structural behaviour change methods of the lifestyle intervention, delivered by multiple sessions, home visits or individual counselling over an extended period of time. There are opportunities for the parent to contact the caregiver at all times. Medium involvement was defined as parents are directly involved in at least two activities within the intervention, delivered in at least four sessions over a period of time of at least 3 months. There are opportunities to consult the intervention team at a set time point in the week. Low involvement was defined as parents are directly involved in at least one occasion/session and are approached in an indirect way during a period of at least 3 months. Additionally, unclear involvement was defined as based on the report of parental involvement in the full text; not enough detail is available to code the behaviour aspect of parent involvement, and no further details were available or have been provided by the authors on request.

In the behaviour change taxonomy of Golley *et al*., five behaviour changes processes and 32 BCTs were identified 9. The five processes underpinning behaviour change process are identify and motivate readiness to change, facilitate motivation to change, provide relevant information and advice/behaviour change strategies, build self-efficacy (and independence) and prevent and manage relapse. The 32 BCTs are summarized in Table 3. An instruction manual was available on how to identify the processes and the BCTs. To increase interrater reliability, a pilot test was performed, in which four trials not included in the final review were reviewed. Both in the pilot test and in the final review, two reviewers (JvdK and FK) performed the coding independently. Different outcomes in the coding were discussed and proposed to an expert (FL) in coding the BCTs of Golley *et al*.

**Table 3:** Behaviour change taxonomy and frequency of techniques used in studies within a range of follow-up post-intervention

Processes underpinning behaviour change process	Techniques reported	All studies (*n* = 22)	End of intervention or interim (*n* = 22)		≤1 year post- intervention (*n* = 16)		≥2 years post- intervention (*n* = 10)	
			Effective (*n* = 18)	Ineffective (*n* = 4)	Effective (*n* = 12)	Ineffective (*n* = 4)	Effective (*n* = 6)	Ineffective (*n* = 4)
Identify and motivate readiness to change	Provide general information on behaviour-health link	21	**17**	4	**11**	4	**6**	4
	Provide information consequences	11	9	2	5	2	**5**	2
	Provide information about others' approval	0	0	0	0	0	0	0
	Provide general encouragement	4	3	1	1	1	3	0
	Motivational interviewing	8	6	2	4	2	4	0
Facilitate motivation to change	Prompt intention formation	12	10	2	7	2	**5**	2
	Prompt specific goal setting	14	10	4	7	4	3	2
	Prompt self-monitoring of behaviour	14	10	4	7	4	4	4
	Agree behavioural contract	9	8	1	2	1	4	0
Provide relevant information and advice/behaviour change strategies	Provide instruction	15	**12**	3	8	3	**5**	4
	Anticipatory guidance	4	2	2	2	2	0	0
	Tailored or personalized delivery	13	**12**	1	7	1	**5**	1
	Environmental restructuring	12	**11**	1	7	1	3	2
	Feeding practices	15	**13**	2	8	2	**5**	2
	Parenting skills: generic	15	**12**	3	**10**	3	3	3
	Parenting skills: specific to lifestyle behaviours	14	**11**	3	7	3	3	3
	Time management (including planning)	2	1	1	1	1	0	1
	Provide contingent rewards	14	**11**	3	**9**	3	3	0
	Teach to use prompts/cues	6	5	1	2	1	2	1
Build self-efficacy (and independence)	Set graded tasks	4	4	0	4	0	1	0
	Model/demonstrate the behaviour	7	5	2	4	2	2	2
	Provide performance feedback	10	8	2	6	2	4	1
	Prompt practice	8	7	1	6	1	2	1
	Provide opportunities for social comparison	5	4	1	4	1	2	1
	Plan social support/social change	17	**14**	3	**10**	3	**6**	2
	Prompt identification as role model/position advocate	11	10	1	8	1	4	2
	Prompt self-talk	0	0	0	0	0	0	0
Prevent and manage relapse	Prompt barrier identification	15	**14**	1	**12**	1	4	1
	Prompt review of behavioural goals	8	6	2	5	2	3	0
	Use of follow-up prompts	5	2	3	1	3	2	1
	Relapse prevention	12	10	2	8	2	4	1
	Stress management	9	8	1	6	1	3	1

Techniques in bold were more frequently identified in effective weight control interventions.

To determine which type of intensity of parent involvement and BCTs were effective, studies were categorized as effective or ineffective based on child weight status and cross tabulated with the intensity of parental involvement and BCTs used in the intervention. A study was classified as supporting effectiveness of the intervention, if it showed a significant change in an objectively measured (not self-reported) variable of obesity (e.g. BMI *z*-score or percent overweight). Due to heterogeneity in both the multi-component interventions and study design, study data were not pooled and results are presented in a narrative form.

## Results

### Study description

Table 1 summarizes 24 studies on health promotion or lifestyle interventions, which aimed to reduce or control body weight and change dietary behaviour in young children in Europe. Eight studies targeted the primary prevention and 16 studies targeted the treatment of overweight and obese children. Individual counselling, group sessions with child and parent, or written materials were the top three modes of intervention delivery. Seven studies primarily investigated obese children only and nine studies primarily investigated overweight and obese children. Long-term treatment effects are available from most intervention trials, with follow-up ranging from 6x months up to 5 years post-tertiary prevention.

**Table 1:** Study characteristics of trials on parental involvement in weight control interventions in children aged 0–12 years in the European Union

Study, country and risk of bias	Design and participants	Data points (duration)	Setting and intervention	Parental involvement	Behaviour change component	Summary of results of primary outcomes
	Primary prevention of obesity						
	*Tigerkids* 20 Germany Low to unclear risk Consort: 15 out of 26	Cluster randomized controlled trial children from German Kindergarten day-care centres Age at baseline: 3–6 years	Baseline and 6 months post-intervention, *N* = baseline/follow-up Intervention: 838 out of 866 Control: 466 out of 463	Setting: school-based Intervention: school year promoting healthy lifestyle by trained teachers	Low intensity Two information evenings. Parents were informed with newsletters providing messages on health related behaviour. An Internet platform was offered.	Primary agent of change: child, kindergarten, parent Health education model five behaviour techniques reported spanning four out of five behaviour change process steps	Prevalence of overweight and obesity not statistically different Summary: 6 months (post-intervention): not effective
	*Crete Health Education Program* 21,22 Crete, Greece Unclear risk of bias Consort 15 out of 26	Longitudinal controlled clinical trial First grade primary school children Intervention: children 1–6th grade Age at baseline: 5.5–6.5 years	Baseline, 3 years, 6 years (end of intervention), 4 years post-intervention *N* = baseline/follow-up Intervention: 602/^*^/^*^/284 Control: 444/^*^/^*^/257 ^*^During 3 and 6 years follow-up, subsamples of participants were analysed (3 year: *n* = 471 and 6 year: *n* = 831 total both groups)	Setting: school-based Intervention: teacher-delivered health and nutritional component, physical education sessions. Workbooks covering dietary issues, physical activity and fitness. Control: no health promotion intervention	Low intensity Annual meetings, parents were given a file containing their child's medical screening results. Nutritional information booklets. Workbook exercises by pupils and their parents	Primary agent of change: child, school, parent Health education model five behaviour techniques reported spanning four out of five behaviour change process steps	Three-year follow-up: control group had a significantly higher change in mean body mass index (BMI) than intervention group (adjusted mean gain 1.8 kg m^−2^ vs. 0.7 kg m^−2^, *P* < 0.001) and suprailiac skin-fold (2.9 vs. 0.8 mm, *P* < 0.05) 6-year follow-up (end of intervention): in the intervention group larger change in BMI (+3·68 vs. +4·28 kg m^−2^ for BMI +2·97 *P* < 0.05 vs. +4·47 mm for biceps skin-fold *P* < 0.001) 10-year follow-up (4 years post-intervention): intervention group: lower average BMI (by 0.7 kg m^−2^, SE 0.28, *P* < 0.019) compared with control group. No differences in the prevalence of overweight between the two groups Summary: 3 years: effective 6 years (end of intervention): effective 4 years (post-intervention): effective
	*Fit von Klein auf* 24–27 Germany Unclear risk of bias Consort: unclear to determine	Cluster randomized cross-over study School children from Kindergarten Age at baseline: 4.6 years (±0.4 years)	Baseline, 1 year (end of intervention), 1-year post-intervention *N* = baseline/follow-up Intervention: 426/?/? Control: 401/?/?	Setting: school based Intervention: teacher-delivered. health tool kit (physical activity, nutrition, stress management), record card of physical exercises for care givers in the kindergarten. Control: no intervention	Low intensity Parent evenings on the theme ‘healthy diet for preschoolers’ and an evening on food and health conducted by a psychologist and a nutritionist.	Primary agent of change: child, school, parent Health education model seven behaviour techniques reported spanning five out of five behaviour change process steps	At 12 months BMI deviation scores (BMI-SDS) of the intervention group declined from 0.08 ± 1.02 (7.2% overweight, 4.4% obese) at baseline with 0.04 SDS, BMI-SDS. Control increased BMI-SDS 0.11 (*P* < 0.001) 1 year after the intervention the BMI-SDS declined further with 0.03 SDS. Summary: 1 year (end of intervention): effective 1 year (post-intervention): effective
	*Lekker Fit* 28–29 The Netherlands Rotterdam Low risk of bias Consort: 19 out of 26	Cluster RCT Primary school children Age at baseline: Grades 3–5: Intervention: 7.7 (1.0) years Control: 7.8 (1.0) years Grades 6–8: Intervention: 10.8 (1.0) years Control: 10.8 (1.0) years	Baseline and 1 year after start of the programme *N* = baseline/follow-up Intervention: 1,240 out of 1,149 (92%) Control: 1,382 out of 1,267 (91%)	Setting: school based Intervention: increased physical activity over the school year and an educational programme on healthy living Control: usual curriculum	Low intensity Homework assignments and fitness score card, written information on the intervention and inviting them for a health promotion gathering at the beginning of the school year	Primary agent of change: child, school, parent Theory of planned behaviour and the ecological model of Egger/Swinburn six behaviour techniques reported spanning three out of five behaviour change process steps	One year after the start of the programme: significant positive intervention effects were found for percentage overweight children (odds ratio [OR] 0.53; 95% CI 0.36–0.78), waist circumference (−1.29 cm; 95% confidence interval [CI] −2.16–−0.42 cm) and 20 m shuttle run (0.57 laps; 95% CI 0.13–1.01 laps) among pupils of grades 3–5 (6–9 year olds). No significant effects were found for BMI or for grades 6–8 (9–12 year olds). Summary 1 year after start of the programme: effective
	*Gezond Gewicht Overvecht* 30 The Netherlands Utrecht Low-unclear risk of bias Consort: 17 out of 26	Non-randomized (quasi-experimental) study Youth (aged 0–19 years) and their community	Baseline (2004/2005), 5 year after start of the intervention (2010) *N* = baseline/follow-up Intervention: 04/05: *n* = 791 05/06: *n* = 830 06/07: *n* = 777 07/08: *n* = 699 08/09: *n* = 419 Total: *n* = 3,532 Control: *n* = 4,163	Setting: district health programme community based Intervention: multiple interventions. Joint initiative of council, health care and municipal organizations Control district: no health promotion intervention	Could not be clearly extracted	No assessment made	Prevalence of overweight (including obesity) in 4–12 year olds was significantly reduced from 26% in 2004/2005 to 20% in 2008/2009 (OR = 0,85 [0,77–0,94]) Summary: 5 years after start of the intervention: effective
	*Epode (Fleurbaix–Laventie Ville Sante Study)* 31–32 France unclear to high risk of bias Consort: 14 out of 26	Cross-sectional study (1992 and 2000 data) Observational study (2002–2004 data) Schoolchildren Age at baseline: 5–12 years old	Baseline (1992), 2000, 2002, 2003, 2004 *N* = sample size Intervention: 1992: *n* = 804, 2000: *n* = 601, 2002: *n* = 515, 2003: *n* = 592 2004: *n* = 633, Control: 2004 out of 2005: *n* = 349	Setting: School/community Intervention: 1. (1992–1997) A school-based nutrition information programme 2. (1997–2002) Every 2 years, health survey families 3. (2002–2007) family-oriented advice on healthy living Control: no intervention	Could not be clearly extracted	No assessment made	(1992–2000) An increase in BMI and height in both boys and girls were observed. Girls: increase in obesity 1.6 to 4.4% (*P* < 0.03) increase in overweight 14.1–18.6% (*P* < 0.11) Boys: increase in overweight 13.8 to 20% (*P* = 0.03) Over a period from 2000 to 2004: Compared with 2002, the age-adjusted OR for overweight in the intervention town was significantly lower in 2003 and 2004 (but for girls only). In the 2004 school year, the overweight prevalence was significantly lower in the intervention town (8.8%) than in the comparison towns (17.8%, *P* < 0.001) Summary 12 years after start of the programme: effective
	Both prevention and treatment of overweight and/or obesity						
	*OKIDO* 33–34 The Netherlands Unclear risk of bias Consort: 15 out of 26	Non-randomized (quasi-experimental) study Primary school children, grade 3–5 Age at baseline: 7–10 years old	Baseline, 4 months (end of intervention), 4 months and 5 years post-intervention *N* = baseline/follow-up Intervention: 129/124/93 (72%) Control: 101/96/74 (73%)	Setting: school and family Intervention: schools: three class sessions on diet and physical activity and a ‘project corner’ in school for children. Family: a family course for parents and overweight children. Control schools: no intervention	School: low intensity Family: medium intensity Only parents of obese or overweight children were directly involved in family intervention. All children: Parents received written health information and child's weight status.	Primary agent of change: child, school, parent Theory of planned behaviour Family: 10 behaviour techniques reported spanning five out of five steps School: four techniques spanning two out of five processes	Effects of both family and school intervention: At 4 months: BMI-SDS increase less in intervention group vs. control (*P* = 0.038), not maintained at 5 years Summary 4 months (end of intervention): not effective 4 months (post-intervention): not effective 5 years (post-intervention): not effective
	*Kiel Obesity Prevention Study: KOPS* 35–38 Germany Unclear risk of bias Consort: 14 out of 26	Cluster-sampled quasi-randomized crossover trial nested in a cohort School children Age at baseline: 5–7 years Family-based intervention: Non-randomized open clinical trial	Baseline, 1 year (end of intervention), 3-year post-intervention *N* = baseline/follow-up School intervention: Intervention: 440/?/345 Control: ?/?/1,419 Family intervention: Intervention: 368/92 (25%) (discontinued at 1-year follow-up)	Setting: school and family Intervention: school intervention: nutritional education by nutritionist and trained teacher. Family intervention: individual counselling by a nutritionist over a period of 3 months. Additional 6 months sports programme	Low intensity School intervention: Health promotion was aimed at school children and their parents. A parent evening for education at school. Family intervention: 3–5 home visits with a nutritionist within a period of 3 months	Primary agent of change: child, school, parent No theory of behaviour specified Family and school: eight behaviour techniques reported spanning three out of five behaviour change process steps	One-year follow-up Significant effects in percentage fat mass of overweight children (increase by 3.6 vs. 0.4% per year without and with intervention, respectively; *P* < 0.05) No differences in BMI between control and intervention schools Significant effects on the age dependent increases in median triceps skin-folds (from 10.9 to 11.3 mm in ‘intervention schools’ vs. from 10.7 to 13.0 mm in ‘control schools’, *P* < 0.01) 3 years post-intervention Intervention had no effect on mean BMI. No significant difference in prevalence of overweight and obesity. Summary 1 year (end of intervention): effective 3 year (post-intervention): not effective
	Treatment of overweight and/or obesity						
	SCOTT 39–40 UK Low risk of bias Consort: 18 out of 26	Randomized controlled trial Overweight children Age at baseline: mean 8.5 (5–11 years)	Baseline, 6 months (end of intervention) and 6 months (post-intervention) *N* = baseline/follow-up Intervention: 69/49/45 (65%) Control: 65/48/42 (63%)	Setting: outpatient hospital clinic Intervention: eight individual appointments Family centred, lifestyle monitoring, aimed at behavioural change in nutrition, activity and weight control Control: standard care	High involvement eight sessions were for child and parent together. Parents had one separate parental session discussing their skills and exploring parental concerns	Primary agent of change: child and parent No theory of behaviour specified 17 behaviour techniques reported spanning five out of five behaviour change process steps	The intervention had no significant effect compared with standard care on BMI *z*-score from baseline to 6 months and 6 months post-intervention. BMI *z*-score decreased significantly in both groups from baseline to 6 and 6 months post-intervention. Summary 6 months (end of intervention): not effective 6 months (post-intervention): not effective
	*Magnificent kids* 41–42 Finland Unclear risk of bias Consort: 19 out of 26	Randomized controlled trial Obese children Age at baseline: mean 8 years (standard deviation [SD] 0.8, 6.6–9.7 years)	Baseline, 6 months (end of intervention), 6 months post-intervention, 1,5 and 2.5 years post-intervention, *N* = baseline/follow-up Control: 35/34/35/35/34 (97%) Intervention: 35/34/34/34/34 (97%)	Setting: family-based in school health care Intervention: Control: routine treatment. Intervention: family-based group treatment sessions including nutrition education, physical activity education and behavioural therapy	High intensity Control: information booklets Intervention: 15 sessions of 90 min. group treatment for parents and children. Group sessions were held separately for children and parents	Primary agent of change: parent Cognitive behavioural and solution oriented therapy 15 behaviour techniques reported spanning five out of five behaviour change process steps	In the intervention group, children lost more weight for height (6.8%) than children receiving routine counselling (1.8%) (*P* < 0.001). The respective decreases in BMI were 0.8 vs. 0.0 (*P* < 0.003) and in BMI-SDS 0.3 vs. 0.2 (*P* < 0.022) 6 months post-intervention, small but significant changes in weight for height and BMI were found. No significant differences between treatment arms in 2- or 3-years follow-up visits Summary 6 months (end of intervention): effective 6 months (post-intervention): effective 1,5 year (post-intervention): not effective 2,5 year (post-intervention): not effective
	*Mi Piace Piacermi* 43 Italy Unclear risk of bias Consort 15 out of 26	Longitudinal observational clinical study Obese children with parents Age at baseline: 8.4 years (6.1–11.9 years)	Baseline, 10 weeks (end of intervention), 5 years (post-intervention) *N* = baseline/follow-up Intervention: 31/22/20 (65%)	Setting: outpatient hospital clinic Intervention: a cognitive-behavioural lifestyle multidisciplinary programme. eight Follow-up visits over the course of 3 years	High intensity Treatment programme activities are proposed to children and their parents, sometimes together and sometimes separately	Primary agent of change: parent cognitive behavioural and transtheoretic model of Prochaska and Di Clemente 16 behaviour techniques reported spanning five out of five behaviour change process steps	In subjects who completed the 5-year follow-up, BMI-SDS was 4.23 ± 0.71 at baseline and 2.74 ± 0.85 at follow-up. Adjusted BMI was 54.7% ± 9.0 at baseline and 43.2% ± 17.3 at the last visit. Both reductions were highly significant Waist circumference decreased. Summary 10 weeks (end of intervention): effective 5 years (post-intervention): effective
	*Obeldicks mini* 44–45 Germany Unclear risk of bias Consort: 15 out of 26	Pre-test/post-test design Obese children Age at baseline: 6.1 ± 1 year (4–7.9 years)	Three months before baseline, baseline, 1 year (end of intervention), 3 years (post-intervention) *N* = baseline/follow-up Intervention: 103/84/64/50 (60%)	Setting: clinic for child and youth health care Intervention: based on diet, exercise and behaviour therapy including individual psychological care of the child and parents. Multidisciplinary team	High intensity Separate parent groups. 13 monthly 1,5-h group sessions for parents. Individual care every 2 months 30 min. Exercise sessions with children every month	Primary agent of change: parent Cognitive behaviour and system therapy 24 behaviour techniques reported spanning five out of five behaviour change process steps	The mean SDS-BMI reduction was 0.46 ± 0.35 (*P* < 0.001). 3 years after end of intervention, the achieved weight loss sustained Summary 1 year (end of intervention): effective 3 year (post-intervention): effective
	*Fitoc* 46–50 Germany Unclear to high risk of bias Consort: 11 out of 26	Non-randomized clinical study Obese children Age at baseline: mean 10.5 years	Baseline, 8 months (end of intervention), 10 months post-intervention, 2^2/3^ year post-intervention *N* = baseline/follow-up Intervention: 496/461/297/137 (28%) Control: 35/35/65/no control in final follow-up	Setting: outpatient university clinic/sports centre Intervention: regular physical exercise plus comprehensive dietary and behavioural education Controls: no intervention	*Medium intensity* Seven separate parents evenings every 4 to 6 weeks, regular nutrition discussions for parents and children	Primary agent of change: parent and child No theory of behaviour specified 14 behaviour techniques reported spanning four out of five behaviour change process steps	After 8 months BMI as well as BMI-SDS decreased in both sexes (*P* < 0.001). In controls, BMI increased (*P* < 0.001) and BMI-SDS remained constant. Ten months post-intervention: Significant improvements in BMI-SDS measured from baseline (*P* < 0.001) Summary 8 months (end of intensive phase intervention): effective 10-month post-intervention: effective 2^2/3^ year post-intervention: not effective
	TAKE 51,52 Switzerland Unclear risk of bias Consort: 17 out of 26	Randomized controlled trial Overweight children Age at baseline: 10 years (range 8–12 years)	Baseline, 9 months (end of intervention), 4 ^1/3^ years post-intervention *N* = baseline/follow-up Intervention 1 (mother and child): 31/25/20 (65%)/13 Intervention 2 (mother only): 25/12/7/(28%)/14	Setting: outpatient university clinic Intervention: Intervention 1 involved mother and child in cognitive behavioural therapy Intervention 2 involved mother in cognitive behavioural therapy and child in progressive muscle relaxation training.	High intensity Cognitive behavioural therapy for parents only (intervention 2) or for parent and child (intervention 1- parent and child in separate groups) using ‘individual treatment in group approach’ by trained psychologists	Primary agent of change: parent Cognitive behavioural therapy 18 behaviour techniques reported spanning five out of five behaviour change process steps	Both interventions reduced children's percent overweight significantly and equally by the end of intervention. 5-year follow-up: Moderate effects on BMI-SDS (−0,11 4.4%) Summary 9 months (end of intervention): effective 4 ^1/3^ year (post-intervention): effective
	*Obeldicks* 54–55 Germany Risk of bias: unclear to high risk Consort: 13 out of 26	Pre-test/post-test design Obese children Age at baseline: mean 10.5 years (6–16 years)	Baseline, 1 year (end of intervention), 1, 2 and 3 years (post-intervention) *N* = baseline/follow-up Intervention: 170/151/142 (83%)	Setting: outpatient university clinic Intervention: multidisciplinary programme is based on physical exercise (1 year), nutrition education and behaviour therapy for children and parents separately	High intensity six group sessions for parents separately from the children: intensive phase/maintenance phase/follow-up phase	Primary agent of change: parent Cognitive behavioural therapy 27 behaviour techniques reported spanning five out of five behaviour change process steps	The mean reduction of SDS-BMI compared to baseline was 0.41 (95% CI 0.37–0.46) at the end of intervention, 0.40 (95% CI 0.34–0.46) 1 year, 0.41 (95% CI 0.33–0.48) 2 years and 0.48 (95% CI 0.37–0.59) 3 years after the end of intervention, respectively. Summary 1 year (end of intervention): effective 1 year (post-intervention): effective 2 year (post-intervention): effective 3 year (post-intervention): effective
	*Families for Health* 56–57 UK Risk of bias: unclear Consort 16 out of 26	Pre-test/post-test design Families with children who were overweight or obese Age at baseline: 7–11 years	Baseline, 3 months (end of intervention), 6 months and 1.5 years (post-intervention) *N* = baseline/follow-up Intervention 27/22/22/19 (70%)	Setting: community/family Intervention: 12 weekly group sessions of 2.5 h parallel for children and parents by local trained health care professionals	High involvement Parent sessions addressed parenting, lifestyle change, social and emotional development. Parents and children eat mid-session for a snack and an activity.	Primary agent of change: parent No theory specified 18 behaviour techniques reported spanning five out of five behaviour change process steps	BMI *z*-score change from baseline was: −0.18 (95% CI −0.30 to −0.05) at 3 months and −0.21 (−0.35 to −0.07) at 6 months post-intervention and −0.23 (95% CI: −0.42 to −0.03, *P* = 0.027) at 1.5 year post-intervention Summary 3 months (end of intervention): effective 6 months (post-intervention): effective 1.5 years (post-intervention): effective
	*Mend* 58–59 UK low risk of bias Consort: 19 out of 26	Randomized controlled trial Obese children Age at baseline: 10 years (8–12 years)	Baseline, 6 months (end of intervention), 6 months (post-intervention) *N* = baseline/follow-up Control: 56/45/38/ (68%) Intervention: 54/37/42 (70%)	Setting: community/family Intervention: parents and children attended 18 2-h group educational and physical activity sessions held twice weekly in sports centres and schools, Control: waiting list (delayed intervention)	High intensity Sessions for parents and children together, five sessions on behaviour change parents/carers only	Primary agent of change: parent and child Social cognitive theories 21 behaviour techniques reported spanning five out of five behaviour change process steps	Intervention group had a reduced waist circumference *z*-score (−0.37; *P* < 0.0001) and BMI *z*-score (−0.24; *P* < 0.0001) at 6 months when compared with the controls. At 6 months post-intervention in the intervention group reduced their waist and BMI *z*-scores by 0.47 (*P* < 0.0001) and 0.23 (*P* < 0.0001), respectively Summary 6 months (end of intervention): effective 6 months (post-intervention): Effective
	Greece 60–61 Unclear to high risk of bias Consort: 12 out o of 26	Randomized controlled trial Overweight children Age at baseline: 9.2 ± 0.2 years	Baseline, 3 months, 6 months (end of intervention) and 1 year (post-intervention) *N* = baseline/follow-up Intervention (child): 19/18/18/16 (84%) Intervention (parent and child): 23/18/18/16 (70%)	Setting: family based Intervention 1 and 2: a multidisciplinary programme assigned high self-regulation in children, but differed in parental involvement Intervention 1 is child only, intervention 2 is parent and child	Medium intensity In the child-and-parent group, parents participated in the last 10 min of each session, acting as helpers in general	Primary agent of change: parent and child Cognitive behavioural therapy nine behaviour techniques reported spanning four out of five behaviour change process steps	Percent overweight decreased by 4.9 ± 1.4 at 1 year post-intervention (*P* < 0.001); the reduction occurred during the active phase of the treatment (0–3 months) and was maintained thereafter Summary 6 months (end of intervention): effective 1 year (post-intervention): effective
	Sweden 62–63 unclear to high risk of bias Consort: 13 out of 26	Randomized open trial Overweight or obese children Age at baseline: 10 years (8–12 years)	Baseline, 1 year (end of intervention), 1 year (post-intervention) *N* = baseline/follow-up Control: 48/28/27 (56%) Intervention: 45/30/29 (64%) Total: 83/52 (62%)	Setting: family based Intervention: programme aimed at improving food and physical activity habits, changing behaviour and improving self-esteem and weight control Control: standard care	Medium intensity 14 group 1–1.5 h sessions for parents and children over 1 year led by dietician. Parent and child were in separate sessions meeting at the end of session	Primary agent of change: parent Behavioural and solution focused group work 19 behaviour techniques reported spanning five out of five behaviour change process steps	No effects on BMI BMI (kg m^−2^) Intervention 23.1 ± 2.65 Control: 23.0 ± 2.97 *P* = 0.132 BMI 1 year post-intervention: no statistical difference was found between the groups regarding body mass index Summary 1 year (end of intervention): not effective 1 year (post-intervention): not effective
	Iceland 64–65 unclear to high risk of bias Consort: 12 out o of 26	Pre-test/post-test design Obese children and parent Age at baseline: mean age 11.0 years (SD 1.4, range 7.5–13.6 years)	Baseline, 18 weeks (end of intervention), 1 year (post-intervention) *N* = baseline/follow-up Intervention: 84/61 (73%)	Setting: outpatient hospital treatment Intervention: Epstein family-based behavioural treatment: nutritional education, physical activity programme, energy restricted diet, self- monitoring and maintenance of behaviour change	High intensity 12 group and 12 individual (parent and child) treatment sessions (12 weeks delivered over 18 weeks). Individual sessions were 20 min, group meetings 90 min.	Primary agent of change: parent and child No behavioural therapy defined 20 behaviour techniques reported spanning five out of five behaviour change process steps	BMI-SDS at baseline: 3.12 (SD 0.5) Change in BMI –SDS (post treatment) −0.40 (SD 0.3) Change in BMI –SDS (1-year post-intervention) −0.35 (SD 0.3) Summary 18 weeks (end of intervention): effective 1 year (post-intervention): effective
	*Dikke Vrienden Club* 66 The Netherlands Rotterdam Unclear to high o risk of bias Consort: 14 out of 26	Pre-test-post-test design Overweight or obese children Age at baseline: 10.5 years ( 8.0–14.0 years)	Baseline; 3 months (end of intervention), 9 months (post-intervention) *N* = baseline/follow-up 73/70/49 (67%)	Setting: outpatient hospital clinic Intervention: eight children sessions and two parent sessions during the first 12 weeks. Multidisciplinary team. The children are paired into age-matched buddy teams. Follow-up visits	Medium intensity Two separate parent sessions in groups over the course of 12 weeks. Follow-up: parent sessions aimed at prevention of relapse are organized	Primary agent of change: parent and child Cognitive behavioural therapy and operant behavioural therapy 10 behaviour techniques reported spanning five out of five behaviour change process steps	Mean BMI-SDS showed a significant reduction of 0.3 BMI-SDS after the 12-week programme (*P* < 0.0001) The participants achieved a 0.6 BMI-SDS reduction; comparable with a weight loss of 18.7% 9 months post-intervention (*P* < 0.0001). Summary 3 months ( end of intervention): effective 9 months (post-intervention): effective
	*Dikke Vrienden Club* 67 The Netherlands Rotterdam Unclear to high risk of bias Consort 14 out of 26	Pre-test-post-test design with 1-year follow-up Overweight or obese children Age at baseline: 11.0 (±1.6) (8.0–14.9 years)	Baseline; 3 months (end of the 12-week intensive programme), 6 and 9 months *N* = baseline/follow-up 248/238/178/151 (63%)	Setting: outpatient hospital clinic Delivery: as above study Intervention: as above study	Medium intensity Minimum of three separate parent sessions in groups over the course of 12 weeks, as above	Primary agent of change: parent and child Cognitive behavioural therapy and operant behavioural therapy nine behaviour techniques reported spanning five out of five behaviour change process steps	Completers had a mean reduction of 0.42 BMI-SDS 9 months post-intervention (*P* < 0.001). At the start of treatment, 82% (*n* = 202/*N* = 248) of the children were obese, while 40% (*n* = 61/*N* = 151) of the children were obese 9 months post-intervention. Summary 3 months (end of the 12-week intensive programme): effective 9 months post-intervention: effective
	*Door Dik en Dun Stichting Right Step* 68 The Netherlands Unclear to high risk of bias Consort: 13 out of 26	Non-randomized (quasi-experimental) study Overweight or obese children Age at baseline: 10.3 (1.8 years)	Baseline; 4 months (end of the intensive programme) and 8 months (post-intervention) *N* = baseline/follow-up Intervention: 47/46/47 Control: 35/33/31	Setting: paramedic setting Intervention: family treatment aiming for change in lifestyle behaviour, Control: standard care of individual dietetic counselling	High intensity Multidisciplinary team group sessions seven group sessions for parents. Follow-up over the course of 8 months consisting of five family sessions. Website. Two individual family counselling sessions	Primary agent of change: parent and child No theory specified 18 behaviour techniques reported spanning five out of five behaviour change process steps	Significant reduction in percentage of obese children 9 months post-intervention The BMI-SDS of the intervention group was significantly reduced from 2.42 (SD = 0.47) to 2.10 (SD = 0.58) versus 2.62 (SD = 0.54) to 2.54 (SD = 0.53) in the control 9 months post-intervention. Summary 4 months (end of the intensive programme): effective 8 months (post-intervention): effective
	*Weet en Beweeg* 69 The Netherlands Low to unclear risk of bias Consort: 17 out of 26	Randomized controlled trial Obese and overweight children and adolescents Age at baseline: 11.3 years (range 6–18)	Baseline, 1 year (end of intervention), 1 year (post-intervention) *N* = baseline/follow-up Intervention: 33/32/not stated Control: 36/33/not stated	Setting: (municipal) Centre for Family and Youth care Intervention: nutrition and physical activity training and behavioural change through a cognitive behavioural therapeutic approach targeting the family as a whole. Control: standard care	High intensity Parents are targeted in 10 separate parent sessions and involved in all aspects of the therapy in order to support the child make lifestyle changes	Primary agent of change: parent and child Cognitive behavioural theory, ASE-model, stages of change model 14 behaviour techniques reported spanning five out of five behaviour change process steps	The mean BMI-SDS significantly decreased in the intervention group (BMI-SDS decrease 0.27), while the mean BMI-SDS remained the same in the control group (BMI-SDS decrease 0.01). The intervention group could preserve the effect reached during the intervention up to 1 year post-intervention Summary 1 year (end of intervention): effective 1 year (post-intervention): effective

### Quality assessment

The results of methodological quality and risk of bias are reported in Table 1. The three studies with a low risk of bias had a randomized controlled design 28,39. In seven studies, follow-up was reported in one or more publications. Quality assessment of these studies was based on all publications 21–54. In 17 studies, the risk of bias was assessed as unclear or unclear to high. In 8 of these 17 studies, study quality was impaired due to poor study design 32–67. Other common reasons for unclear to high risk of bias in reviewed studies are non-randomization, small sample size and high drop-out. Loss to follow-up increased over the course of the time of post-intervention measurements 43,50.

### Parental involvement

All 24 trials were classified according to the criteria of high, medium or low involvement. Table 1 describes how parents were involved in the intervention. In primary prevention programmes, parents were involved in meetings which were organized by school and by provision of written information materials. Weight loss interventions were mostly delivered in group sessions, in which child and parent were together, or were in separate sessions, occasionally interacting. Table 2 shows the intensity of parental involvement and effectiveness of the studies at the end of the intervention or at interim and 2 years post-intervention. In prevention studies, only low intensity parental involvement was identified, whereas in effective treatment studies medium and high parental involvement were identified most frequently.

**Table 2:** Intensity of parental involvement and effectiveness at end of intervention or at interim and 2-year post-intervention

Type of studies	Intensity of parental involvement	End of intervention or at interim			>2-year post-intervention			No data (*n* = 12)
		Studies (*n* = 24)	Effective studies (*n* = 18)	Ineffective studies (*n* = 6)	Studies (*n* = 24)	Effective studies (*n* = 8)	Ineffective studies (*n* = 4)	
	Primary prevention	Low parental involvement	4	3	1	4	1	1	2
		Unclear parental involvement	2	2	0	2	2	0	0
	Treatment	Low parental involvement	1	0	1	1	0	1	0
		Medium parental involvement	6	4	2	6	1	1	4
		High parental involvement	11	9	2	11	4	1	6

### Behavioural change techniques

Using the taxonomy of BCTs, 22 studies were coded 7. The interventions of EPODE and Utrecht Overvecht Gezond Gewicht were not coded, due to the complexity of these multiple interventions and the inability to describe clearly parental involvement 30–69. Table 3 lists the five behaviour change processes and the 32 techniques used to code intervention description. We scored the results of effective and ineffective interventions at the end of the intervention or at interim (*n* = 22), ≤1-year post-intervention (*n* = 16) and ≥2 years post-intervention (*n* = 10).

Sixteen studies reported all five behaviour change process steps, four studies reported four out of five and two studies reported three out of five. All treatment studies included a minimum four behaviour change process steps. In total, 310 techniques were reported in all studies (*n* = 24). Primary prevention studies scored 23 BCTs and the treatment studies scored 287 BCTs. Treatment studies used more BCTs than the primary prevention studies.

Table 4 shows the descriptions found in the included studies about the BCTs parenting skills generic and parenting skills specific to lifestyle. Parenting skills generic and specific are reported in ≥50% in effective studies at the end of the intervention or at interim, ≤1-year post-intervention and ≥2 years post-intervention.

**Table 4:** Parenting skills generic and specific to lifestyle

Parenting skills generic:	Parenting skills specific to lifestyle:
	Skills to use praise and adequate reward to reinforce the children, how to apply positive enforcement, importance of using praise with children	Enforcing dinner table rules in a positive way: taking the time to eat, no eating in front of the TV, any food on the table is offered to all members of the family, keeping stress away from the family table
	Increasing the quality of authority and control of the parent over the child, instruction on how to set boundaries for the child	Giving the good example of modelling physical activity
	Stimulating and supporting the child to deal with bullying	Providing structure in frequency of meals
	Skills training to support adequately the child, skills to help the child deal with negative emotions	Controlling portion sizes of meals in a positive way, helping the child to differentiate between hunger and craving
	Learning to provide an open environment for communication, listening to each other, sharing ideas and opinions, consulting model	Undertaking activities together (fun and play)
	Teaching parents how to deal with different children in one family	Create awareness of who is responsible for achieving good physical activity and good food habits
	Changing interaction patterns between parents and child by teaching them how to support the child instead of controlling them	Providing a supportive environment in terms of food availability and accessibility
	Addressing important topics with parents/carers to help them implement these topics at home	Practices used to control the child's dietary intake and to monitor the child's food intake
	Family rules family rewards, nurturing our families nurturing ourselves	Skills to implement anti-obesogenic strategies
	Skills to implement and maintain behaviour change	Developing family rules that will support the development of healthy lifestyle behaviours within the home
Training in assertiveness	Enabling mothers to cope with stigmatization of obesity in their offspring
	Skills to modify behaviour step by step

#### Behavioural change techniques in long-term effective studies

From the 32 BCTs in the studies, 10 techniques scored ≥ 11 in effective studies at the end of intervention or interim (*n* = 22) (Table 3). More than 2 years post-intervention, the most frequently reported techniques in effective studies were provide general information on behaviour-health link (six out of six studies), provide information consequences (five out of six studies), prompt intention information (five out of six studies), provide instruction (five out of six studies), tailored or personalized delivery (five out of six studies), feeding practices (five out of six studies) and plan social support social change (six out of six studies).

## Discussion

We systematically reviewed the intensity of parental involvement and BCTs aimed at parents in long-term childhood weight control intervention using a taxonomy to describe studies in terms of intervention content. This review showed that in all prevention studies, the intensity of parental involvement was identified as low, whereas in treatment studies the intensity of parental involvement was identified as low, medium or high. No conclusions could be drawn concerning the low intensity of parental involvement in prevention programmes, due to the small number of the included primary effective prevention studies at the end of the intervention or at interim (*n* = 3) and >2 years post-intervention (*n* = 1). In the included treatment studies measured at the end of intervention or at interim (*n* = 13) and measured >2 years post-intervention (*n* = 5), medium and high intensity of parental involvement were identified most frequently. This finding suggests that the level of intensity of parental involvement is an important issue in weight control interventions.

We identified less reported BCTs in primary prevention studies compared with treatment studies. The BCTs most frequently identified differed per time of follow-up. The most frequently identified BCTs ≥2 years post-intervention were provide general information on behaviour-health link, provide information consequences, prompt intention formation, provide instruction, tailored or personalized delivery and plan social support/social change. In contrast to ≤1 year post-intervention, parenting skills generic was infrequently identified ≥2 years post-intervention.

The results of this systematic review are in line with the findings of the study by Hingle *et al*., in which direct approaches to engage parents were more likely to result in positive outcomes than indirect methods 70. Within the reported techniques of parental barriers in the treatment of childhood obesity, we found similarities with the study by Pocock *et al*. 11. The most common theme related to parental perception is lack of time, which acts as a barrier to child exercise and healthy diet 11. In our review, however, the BCT time management was reported only twice in all included studies. For that reason, we recommended attention to time management in developing or implementing weight control interventions.

The strength of this review is the scope of reviewing long-term multi-component lifestyle interventions with a parental, a behavioural change and a nutritional component in childhood obesity by identifying the role of parental involvement in the European Union. We specifically studied sustainable effects of parental involvement on weight control. Effectiveness of interventions was identified both using a classification system of intensity of parental involvement and a taxonomy to describe studies in terms of intervention content. Also, parenting skills general and parenting skills specific to lifestyle were identified explicitly.

However, this review has several limitations. Due to the multi-component design of the interventions under study, change in energy-related behaviour or weight can be inferred as being caused or influenced by many factors such as physical activity and sedentary behaviour, and not by parental involvement only. In our opinion, interventions should focus on both energy intake and energy expenditure. However, the taxonomy by Golley *et al*. does not identify physical activity as a specific BCT. After finishing the process of coding, a refined version of the manual of the CALO-RE taxonomy was published 71. The specific focus of the CALO-RE taxonomy is changing physical activity and healthy eating behaviours. This taxonomy may offer additional value to prevent childhood obesity since both aspects in energy balance (intake and expenditure) are included. Another limitation is the lack of identifying intensity of the various BCTs. Unfortunately, the taxonomy of Golley *et al*. identifies underpinning processes and BCTs in behaviour change only. In addition to the use of the taxonomy of Golley *et al*., we assessed intensity of parental involvement by classifying this involvement into low, medium or high parental involvement. However, it is unknown whether the value of each separate BCT in the context of the multi-component intervention is equal in outcome of effectiveness. Therefore, insight in the intensity of BCTs is needed. Furthermore, more clinical relevant cut-off points may be needed to determine whether an intervention is effective.

Our process of coding behaviour change was based on manuals and description of the intervention, and limited by the quality of reporting in the publications of the studies. In the publications, we found no details on differences between description and implemented intervention in practice. Therefore, the assumption was made that the intervention was implemented in the exact same way as stated by the description. However, discrepancies in coding might be possible for encodings from different publications. Further, we included only studies published in English, Dutch or German. Many interventions in national or regional settings are published in other languages such as French or Spanish. These language restrictions limited the extent of the publications reviewed and had consequences for viewing all of the literature in the cultural diversity of Europe.

## Conclusion

This systematic review provides a detailed overview of the intensity of parental involvement and BCTs in childhood weight control interventions in children (aged 0–12 years) in the European Union. Low parental involvement was identified in prevention studies, whereas medium and high parental involvements were frequently reported in long-term effective treatment studies. In treatment studies, BCTs were identified more frequently compared to prevention studies. Furthermore, the BCTs most frequently identified differed per time of follow-up. The analysis of parenting skills, generic and specific to lifestyle behaviour offers additional content information and was identified as being high in effective weight control interventions specific during the intervention until ≤1 year post-intervention.

## Conflicts of interest statement

The authors declare there is no conflict of interest.
